# Book Reviews

**Published:** 1897-07

**Authors:** 


LIPPINCOTT'S MEDICAL DICTIONARY. A Complete Vocabulary of the Terms used in Medicine and the Allied Sciences. With their Pronunciation, Etymology and Signification, including much Collateral Information of a Descriptive and Encyclopedic Character. Prepared on the basis of Thomas's Complete Medical Dictionary. By RYLAND W. GREENE, A. B. With the editorial collaboration of John Ashhurst, Jr., M. D., LL. D., Barton Professor of Surgery and Professor of Clinical Surgery in the University of Pennsylvania; George A. Piersol, M. D., Professor of Anatomy in the University of Pennsylvania and Joseph P. Remington, Ph. M., F. C. S., Professor of Theory and Practice of Pharmacy in the Philadelphia College of Pharmacy. Philadelphia: J. B. Lippincott Company. 1897.

Though there has been a wealth of dictionaries issued of late there is always room for good ones near the top. The one before us takes rank with the good ones and as such will command the attention of the profession. One of the distinguished features of the book is its convenient size—a point of much importance. Another one is its plain letter-press. The words defined are printed in heavy-faced type and the definitions in long primer letter. Some of the definitions have been very much elaborated, though through systematic abbreviation and condensation space has been economised without detracting from the usefulness of the book.

One of the most difficult problems connected with dictionary making is pronunciation, but in this work a comprehensive system has been adopted that is at once intelligible and in accord with the most modern philology. We are at a loss, however, to understand why in this day and age a modern dictionary issued by modern men should cling to the old-fashioned diphthongs that are fast becoming obsolete. The contrast between this book and Gould's, which is one of the best, is in this respect remarkable. It is a great pity that medical philologists cannot agree in convention upon a uniform practice in regard to spelling and pronunciation.

In spite of this and some minor defects we end as we began by commending the volume as entitled to a place in the front rank.

---

International Clinics. A Quarterly of Clinical Lectures on Medicine, Neurology, Surgery, Gynecology, Obstetrics, Ophthalmology, Laryngology, Pharyngology, Rhinology, Otology and Dermatology; and specially prepared articles on treatment. By professors and lecturers in the leading medical colleges of the United States, Germany, Austria, France, Great Britain and Canada. Edited by Judson Daland, M. D., (Univ. of Penna.) Philadelphia, Instructor in Clinical Medicine and Lecturer on Physical Diagnosis in the University of Pennsylvania; Assistant Physician to the Hospital of the University of Pennsylvania, etc.; Fellow of the College of Physicians of Philadelphia. J. Mitchell Bruce, M. D., F. R. C. P., London, England, Physician to, and Lecturer on, the Principles and Practice of Medicine at the Charing Cross Hospital. David W. Finlay, M. D., F. R. C. P., Aberdeen, Scotland, Professor of Practice of Medicine in the University of Aberdeen; Physician to, and Lecturer on, Clinical Medicine in the Aberdeen Royal Infirmary, etc. Volume I. Seventh series. April, 1897. Octavo, pp. xii.—344. Philadelphia: J. B. Lippincott Co. 1897.

This volume is contributed to by a number of distinguished men in America and Europe. There are fifteen lectures on treatment, ranging from appendicitis to pneumonia; five articles on medicine; six lectures on neurology; ten contributions on surgery; four on obstetrics and gynecology; one lecture in the department of ophthalmology; three in that of laryngology, pharyngology, rhinology and otology; and two lectures on the topic of dermatology. It will thus be observed that the range of the volume is wide and its teaching comprehensive in character. One of the advantages this series of clinics possesses is originality. Each lecturer turns on the light from his own personal standpoint and so makes his subject interesting even if incomplete. In order to make the series valuable for a reference library it should be unbroken; hence the publishers should be careful to avoid any breaks in transmitting it to those journals they have selected as recipients of the clinic.

